# Long COVID and Significant Activity Limitation Among Adults, by Age — United States, June 1–13, 2022, to June 7–19, 2023

**DOI:** 10.15585/mmwr.mm7232a3

**Published:** 2023-08-11

**Authors:** Nicole D. Ford, Douglas Slaughter, Deja Edwards, Alexandra Dalton, Cria Perrine, Anjel Vahratian, Sharon Saydah

**Affiliations:** ^1^Coronavirus and Other Respiratory Viruses Division, National Center for Immunization and Respiratory Diseases, CDC; ^2^General Dynamics Information Technology, Atlanta, Georgia; ^3^Division of Health Interview Statistics, National Center for Health Statistics, CDC.

**Summary**

**What is already known about this topic?**

Long COVID includes a wide range of ongoing symptoms that can last for weeks, months, or years following SARS-CoV-2 infection.

**What is added by this report?**

Prevalence of long COVID among noninstitutionalized U.S. adults aged ≥18 years decreased from 7.5% (95% CI = 7.1–7.9) during June 1–13, 2022 to 6.0% (95% CI = 5.7–6.3) during June 7–19, 2023 and from 18.9% (95% CI = 17.9–19.8) to 11.0% (95% CI = 10.4–11.6) among adults reporting previous COVID-19. After an initial decline, prevalence remained unchanged beginning January 4–16, 2023. Approximately one quarter of adults with long COVID report significant activity limitations.

**What are the implications for public health practice?**

COVID-19 prevention efforts, including staying up to date with recommended COVID-19 vaccination and planning for long COVID symptom management and health care service needs, remain important.

## Abstract

Long COVID is a condition encompassing a wide range of health problems that emerge, persist, or return following COVID-19. CDC analyzed national repeat cross-sectional Household Pulse Survey data to estimate the prevalence of long COVID and significant related activity limitation among U.S. adults aged ≥18 years by age group. Data from surveys completed between June 1–13, 2022, and June 7–19, 2023, indicated that long COVID prevalence decreased from 7.5% (95% CI = 7.1–7.9) to 6.0% (95% CI = 5.7–6.3) among the overall U.S. adult population, irrespective of history of previous COVID-19, and from 18.9% (95% CI = 17.9–19.8) to 11.0% (95% CI = 10.4–11.6) among U.S. adults reporting previous COVID-19. Among both groups, prevalence decreased from June 1–13, 2022, through January 4–16, 2023, before stabilizing. When stratified by age, only adults aged <60 years experienced significant rates of decline (p<0.01). Among adults reporting previous COVID-19, prevalence decreased among those aged 30–79 years through fall or winter and then stabilized. During June 7–19, 2023, 26.4% (95% CI = 24.0–28.9) of adults with long COVID reported significant activity limitation, the prevalence of which did not change over time. These findings help guide the ongoing COVID-19 prevention efforts and planning for long COVID symptom management and future health care service needs.

## Introduction

Long COVID includes a wide range of ongoing respiratory, neurologic, cardiovascular, and other symptoms that can last for weeks, months, or years following SARS-CoV-2 infection. Estimates of long COVID incidence among nonhospitalized adults with COVID-19 range from 7.5% to 41% (*1*). Long COVID places substantial strain on the health care system (*2*). A retrospective cohort study among eight large integrated U.S. health systems found that SARS-CoV-2 infection was associated with a 4% increase in health care utilization over the 6 months following a positive SARS-CoV-2 test result (*2*). Further, long COVID can have a significant impact on quality of life, functional status, and ability to work (*3*). A study of the 2021–2022 Omicron BA.1/BA.2 wave in Australia found that long COVID was responsible for 74% of the years lived with disability from SARS-CoV-2 infections (*4*).

Some populations might be at increased risk for long COVID, including those who experience more severe acute SARS-CoV-2 infection.* Adults aged ≥50 years are more likely to have severe COVID-19 than are younger persons^†^; however, the risk for long COVID and significant activity limitation by age is not well characterized.

## Methods

CDC analyzed data from the Census Bureau’s Household Pulse Survey (HPS) from June 1–13, 2022 to June 7–19, 2023, with the exception of August 24–September 13, 2022 and November 30–December 8, 2022, when no data were collected. The HPS is a rapidly deployed, cross-sectional national survey with a 2 weeks on, 2 weeks off collection and dissemination approach designed to measure the social and economic effects of COVID-19 on U.S. households.^§^ Long COVID questions were added to the survey beginning June 1, 2022. The HPS sampling frame was derived from the U.S. Census Bureau Master Address File and included all valid addresses with an associated mobile phone number or an email address.^¶^ Respondents reported previous COVID-19 diagnosis** (i.e., ever tested positive for COVID-19 or were told by a doctor or other health care provider they had COVID-19) and current long COVID via an online survey.^††^ Beginning September 14, 2022, participants were asked about significant activity limitation from long COVID (i.e., long-term symptoms significantly reduced ability to carry out day-to-day activities compared with the time before having COVID-19).^§§^

Two-week weighted period prevalence (%) and 95% CIs were estimated for long COVID among those reporting previous COVID-19. In the interest of generating estimates for the overall adult U.S. population, prevalence (with 95% CIs) were also estimated among all adults irrespective of reported prior COVID infection. Significant activity limitation prevalence (with 95% CIs) was estimated among those with long COVID. Two-week weighted period prevalence (with 95% CIs) for long COVID and significant activity limitation were also estimated by age group. Estimates were weighted to adjust for nonresponse, survey coverage, and number of adults per household, and to match Census Bureau estimates of the population by age, sex, race and ethnicity, and educational attainment.^¶¶^ All estimates in these analyses meet the National Center for Health Statistics Data Presentation Standards*** and are publicly available.^†††^ Change in 2-week period prevalence of long COVID and significant activity limitation was evaluated using Joinpoint regression. Joinpoint regression uses permutation tests to identify statistically significant points where linear trends change in direction or magnitude (i.e., joinpoints). The rate of change was tested for each trend to determine whether it was significantly different from zero, and each trend was described in the final model by percentage change (with 95% CIs) for each 2-week survey cycle. All analyses were conducted using Joinpoint (version 5.0; National Cancer Institute). This activity was reviewed by CDC and was conducted consistent with applicable federal law and CDC policy.^§§§^

## Results

**FIGURE 1:**
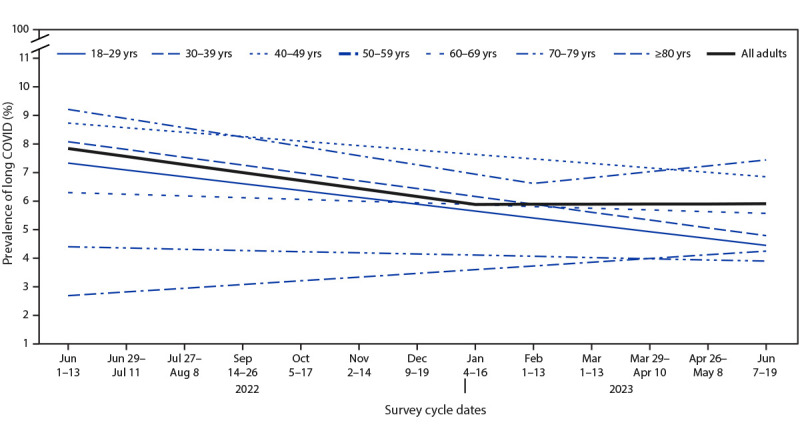
Trend lines for the prevalence of self-reported long COVID among all adults,* by age group — Household Pulse Survey, United States, June 1–June 13, 2022 to June 7–June 19, 2023† **Abbreviation:** HPS = Household Pulse Survey. * Estimate for all adults (slope for June 1–13, 2022 to January 4–16, 2023 = –0.28, p = 0.001; slope for January 4–16, 2023 to June 7–19, 2023 = 0.006, p = 0.95). Estimates of rate of change by age group: 18–29 years (slope for June 1–13, 2022 to June 7–19, 2023 = –0.24, p<0.001); 30–39 years (slope for June 1–13, 2022 to June 7–19, 2023 = –0.27, p<0.001); 40–49 years (slope for June 1–13, 2022 to June 7–19, 2023 = –0.16, p = 0.003); 50–59 years (slope for June 1–13, 2022 to February 1–13, 2023 = –0.32, p = 0.001; slope for February 1–13, 2023 to June 7–19, 2023 = 0.21, p = 0.22); 60–69 years (slope for June 1–13, 2022 to June 7–19, 2023 = –0.06, p = 0.18); 70–79 years (slope for June 1–13, 2022 to June 7–19, 2023 = –0.04, p = 0.40); and ≥80 years (slope for June 1–13, 2022 to June 7–19, 2023 = 0.13, p = 0.13). ^†^ No HPS data were collected during the 2-week period August 24–September 13, 2022, or during November 30–December 8, 2022.

Prevalence of long COVID among all U.S. adults decreased from 7.5% (95% CI = 7.1–7.9) during June 1–13, 2022, to 6.0% (95% CI = 5.7–6.3) during June 7–19, 2023 (Figure 1). From June 1–13, 2022, through January 4–16, 2023, prevalence decreased 0.28% per survey cycle (p = 0.001), then remained stable (0.006% change per cycle, p = 0.95). Statistically significant rates of decline only occurred among adults aged <60 years. Among adults aged 50–59 years, long COVID prevalence decreased 0.32% per survey cycle through February 1–23, 2023 (p = 0.001), then remained stable (0.21% change per survey cycle, p = 0.22).

**FIGURE 2:**
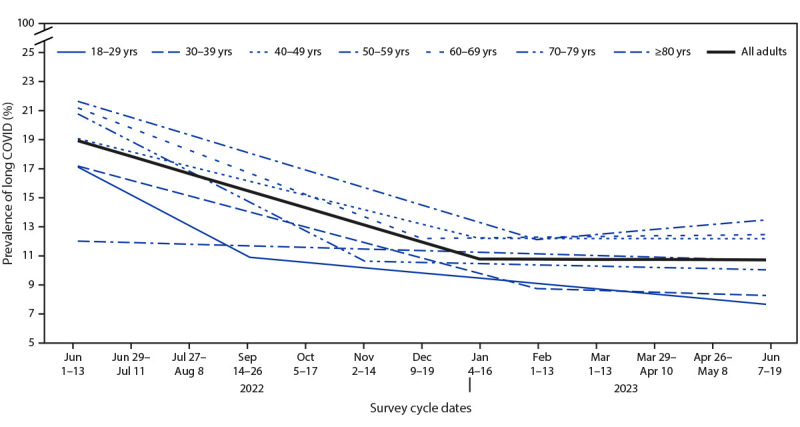
Trend lines for the prevalence of self-reported long COVID among adults with reported previous COVID-19,* by age group — Household Pulse Survey, United States, June 1–June 13, 2022, to June 7–June 19, 2023^†^ **Abbreviation:** HPS = Household Pulse Survey. * Estimate for all adults (slope for June 1–13, 2022 to January 4–16, 2023 = –1.16, p<0.001; slope for January 4–16, 2023 to June 7–19, 2023 = –0.01, p = 0.91). Estimates of rate of change by age group: 18–29 years (slope for June 1–13, 2022 to September 14–26, 2022= –2.07, p = 0.07; slope for September 14–26, 2022 to June 7–19, 2023 = –0.36, p = 0.04); 30–39 years (slope for June 1–13, 2022 to February 1–13, 2023 = –1.05, p<0.001; slope for February 1–13, 2023 to June 7–19, 2023 = –0.12, p = 0.76); 40–49 years (slope for June 1–13, 2022 to January 4–16, 2023 = –0.98, p<0.001; slope for January 4–16, 2023 to June 7–19, 2023 = –0.005, p = 0.98); 50–59 years (slope for June 1–13, 2022 to February 1–13, 2023 = –1.19, p<0.001; slope for February 1–13, 2023 to June 7–19, 2023 = 0.34, p = 0.15); 60–69 years (slope for June 1–13, 2022 to December 9–19, 2022 = –1.50, p<0.001; slope for December 9–19, 2022 to June 7–19, 2023 = 0.05, p = 0.8); 70–79 years (slope for June 1–13, 2022 to November 2–14, 2022 = –2.03, p = 0.04; slope for November 2–14, 2022 to June 7–19, 2023 = –0.09, p = 0.75); and ≥80 years (slope for June 1–13, 2022 to June 7–19, 2023 = –0.11, p = 0.73). ^†^ No HPS data were collected during the 2-week period August 24–September 13, 2022, or during November 30–December 8, 2022.

Among adults reporting previous COVID-19, long COVID prevalence decreased from 18.9% (95% CI = 17.9–19.8) to 11.0% (95% CI = 10.4–11.6) during the study period (Figure 2). Prevalence decreased 1.16% per survey cycle from June 1–13, 2022, until January 4–16, 2023 (p<0.0001), then remained stable (−0.01% change per cycle, p = 0.91). Prevalence of long COVID among adults aged 30–79 years declined through fall or winter, after which it remained stable; the inflection point where long COVID prevalence stabilized varied in timing by age group, ranging from November 2–14, 2022 (adults aged 70–79 years) to February 1–13, 2023 (adults aged 30–39 years and 50–59 years). Among all adults and among those reporting previous COVID-19, long COVID prevalence tended to be lower among the youngest and the oldest age groups (i.e., 18–29 years and ≥60 years).

**FIGURE 3:**
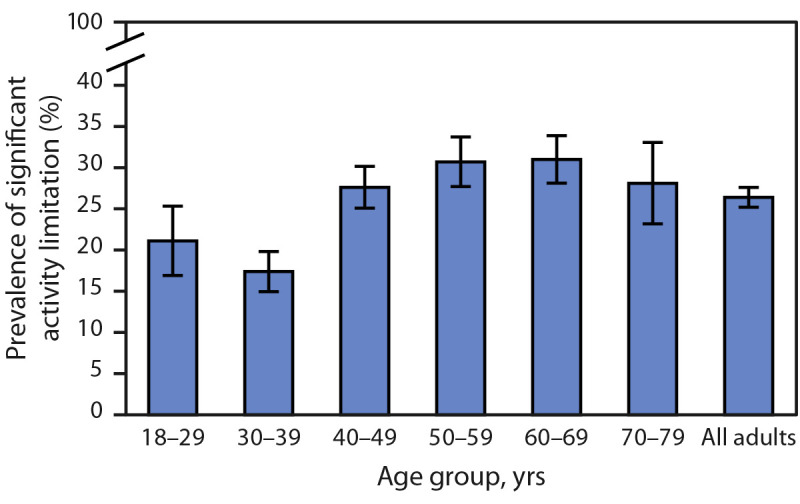
Prevalence of significant activity limitation among adults reporting long COVID* — Household Pulse Survey, United States, June 7–19, 2023 Abbreviation: NCHS = National Center for Health Statistics. * With 95% CIs represented by error bars. Estimates for the adults aged ≥80 years do not meet NCHS Data Presentation Standards and are not included in the figure as a separate group; however, they are included in the estimate for all adults.

During June 7–19, 2023, 26.4% (95% CI = 24.0%–28.9%) of adults with long COVID reported significant activity limitations (Figure 3), the prevalence of which remained stable during the study period (–0.05% change per survey cycle, p = 0.72). No clear pattern emerged for prevalence of significant activity limitation across age groups.

## Discussion

The findings from this analysis of a national sample of U.S. adults indicated that long COVID prevalence decreased from June 1–13, 2022 to June 7–19, 2023. The joinpoint identified during January 4–16, 2023 suggests that, after an initial decline, long COVID prevalence remained unchanged. The decline during the study period might be reflective of decreasing prevalence of SARS-CoV-2 infection,^¶¶¶^ changes in the severity of acute infection,**** interventions offered during acute infection (e.g., antivirals) (*5*), vaccination coverage (*5*), or other factors. Long COVID prevalence has not changed since January 2023, and approximately 1 in 10 adults with previous COVID-19 were experiencing long COVID at the end of the study period, highlighting the ongoing importance of COVID-19 prevention actions, including vaccination.^††††^

Long COVID prevalence among adults tended to be lower in the youngest (18–29 years) and the oldest (≥60 years) age groups, consistent with findings from both U.K. and U.S. studies. In a study of long COVID during the Omicron BA.4/BA.5 surge (June–July 2022), the sex-standardized prevalence of long COVID was lowest among U.S. respondents aged ≥65 years (14.8%, 95% CI = 10.8%–19.9%) and highest among those aged 35–44 years (27.6%, 95% CI = 19.3%–37.8%) (*6*). In the United Kingdom, long COVID prevalence was highest among adults aged 35–49 years.^§§§§^ Lower prevalence of long COVID among older adults might be a consequence of survivor bias, lower prevalence of ever having COVID-19,^¶¶¶¶^ or differences in behavior, such as bivalent vaccination receipt,***** or other^†††††^ self-reported COVID mitigation behaviors (*7*).

More than one in four adults with long COVID reported significant activity limitations during June 7–19, 2023, and prevalence did not change over time. No significant activity limitation prevalence patterns were apparent across age groups. Limited ability to carry out day-to-day activities because of long COVID symptoms can have a significant impact on quality of life, functional status, and ability to work or provide care to others (*3*). Health-related quality of life scores among long COVID patients in the United Kingdom were similar to those of patients with advanced cancers, and 53% reported moderately severe functional impairment, worse than that associated with stroke (*3*). Long COVID in U.S. adults has also been associated with lower likelihood of working full time and higher likelihood of being unemployed (*8*). According to data from the New York State Insurance Fund, 18% of claimants with long COVID could not return to work for more than 1 year.^§§§§§^ The larger economic and societal impact of long COVID could be far-reaching if working-age adults are unable to maintain employment or care for children or aging parents.

### Limitations

The findings in this study are subject to at least three limitations. First, the HPS samples from housing units with at least one matched mobile phone number or email address, and thus is subject to coverage bias. Second, response rate was low for all survey cycles (range = 3.9%–7.0%). Even after weighting and adjustments for coverage and nonresponse, person-level coverage varied by some demographic characteristics. Finally, the survey did not capture duration of symptoms, COVID-19 vaccination status, time since COVID-19 illness, or treatment during acute COVID infection, each of which could influence the reported prevalence of long COVID. Despite these limitations, population-based observational studies, like the HPS, might complement studies based on administrative data by providing insight into experiences of long COVID, including among persons who might not have accessed care.

### Implications for Public Health Practice

After an initial decline during the study period, the prevalence of long COVID has not decreased. The percentage of persons with long COVID who are experiencing significant activity limitations did not change over time. These findings highlight the importance of COVID prevention, including staying up to date with recommended COVID-19 vaccination, and could inform health care service needs planning, disability policy, and other support services for persons experiencing severe activity limitation from long COVID.

## Footnotes

*
https://www.cdc.gov/coronavirus/2019-ncov/long-term-effects/index.html

†
https://covid.cdc.gov/covid-data-tracker/#demographics

§
https://www.census.gov/data/experimental-data-products/household-pulse-survey.html

¶ The HPS sampling frame included approximately 88% of valid addresses. Sampled housing units were contacted by both email and text message if both were available. One respondent from each sampled housing unit answered for themselves. https://www2.census.gov/programs-surveys/demo/technical-documentation/hhp/Phase3-9_Source_and_Accuracy_Week58.pdf

** Previous SARS-CoV-2 infection was ascertained by an affirmative response to the question, “Have you ever tested positive for COVID-19 (using a rapid point-of-care test, self-test, or laboratory test) or been told by a doctor or other health care provider that you have or had COVID-19?”

†† Among respondents reporting previous COVID-19, currently experiencing long COVID was ascertained by affirmative responses to two questions: “Did you have any symptoms lasting 3 months or longer that you did not have prior to having coronavirus or COVID-19? (long term symptoms might include: tiredness or fatigue; difficulty thinking, concentrating, forgetfulness, or memory problems [sometimes referred to as “brain fog”]; difficulty breathing or shortness of breath; joint or muscle pain; fast-beating or pounding heart [also known as heart palpitations]; chest pain; dizziness on standing; menstrual changes; changes to taste/smell; or inability to exercise)” and “Do you have symptoms now?” Respondents who reported no previous COVID-19 were classified as not having long COVID.

§§ When asked, “Do these long-term symptoms reduce your ability to carry out day-to-day activities compared with the time before you had COVID-19?” participants responding “yes, a lot” were classified as having significant activity limitation.

¶¶
https://www2.census.gov/programs-surveys/demo/technical-documentation/hhp/Phase3-9_Source_and_Accuracy_Week58.pdf

***
https://www.cdc.gov/nchs/data/series/sr_02/sr02_175.pdf

†††
https://www.cdc.gov/nchs/covid19/pulse/long-covid.htm

§§§ 45 C.F.R. part 46.102(l)(2), 21 C.F.R. part 56; 42 U.S.C. Sect. 241(d); 5 U.S.C. Sect. 552a; 44 U.S.C. Sect. 3501 et seq.

¶¶¶
https://covid.cdc.gov/covid-data-tracker/#datatracker-home

****
https://covid.cdc.gov/covid-data-tracker/#trends_weeklyhospitaladmissions_select_00

††††
https://www.cdc.gov/coronavirus/2019-ncov/prevent-getting-sick/prevention.html

§§§§
https://www.ons.gov.uk/peoplepopulationandcommunity/healthandsocialcare/conditionsanddiseases/bulletins/prevalenceofongoingsymptomsfollowingcoronaviruscovid19infectionintheuk/3march2022

¶¶¶¶ In the HPS during June 7–19, 2023, 60.6% of adults aged 18–29 years reported ever having SARS-CoV-2 infection compared with 35.7% of adults aged ≥80 years. https://www.cdc.gov/nchs/covid19/pulse/long-covid.htm

***** As of May 10, 2023, bivalent vaccination coverage among adults aged ≥65 years was 43.3% compared with 20.5% among adults aged ≥18 years. https://covid.cdc.gov/covid-data-tracker/#vaccination-states-jurisdictions

††††† Early in the pandemic, self-reported COVID mitigation behaviors including mask wearing, handwashing, physical distancing, crowd and restaurant avoidance, and cancellation of social activities were highest among adults aged >60 years.

§§§§§
https://ww3.nysif.com/en/FooterPages/Column1/AboutNYSIF/NYSIF_News/2023/20230124LongCovid
